# Investigation of Growth Differentiation Factor 15 as a Prognostic Biomarker for Major Adverse Limb Events in Peripheral Artery Disease

**DOI:** 10.3390/jcm14155239

**Published:** 2025-07-24

**Authors:** Ben Li, Farah Shaikh, Houssam Younes, Batool Abuhalimeh, Abdelrahman Zamzam, Rawand Abdin, Mohammad Qadura

**Affiliations:** 1 Department of Surgery, University of Toronto, Toronto, ON M5S 1A1, Canada; benx.li@mail.utoronto.ca; 2 Division of Vascular Surgery, St. Michael’s Hospital, Unity Health Toronto, University of Toronto, 30 Bond Street, Suite 7-076, Toronto, ON M5B 1W8, Canada; 3 Institute of Medical Science, University of Toronto, Toronto, ON M5S 1A1, Canada; 4 Temerty Centre for Artificial Intelligence Research and Education in Medicine (T-CAIREM), University of Toronto, Toronto, ON M5S 1A1, Canada; 5 Heart, Vascular, & Thoracic Institute, Cleveland Clinic Abu Dhabi, Abu Dhabi 112412, United Arab Emirates; 6 Department of Medicine, McMaster University, Hamilton, ON L8S 4L8, Canada; 7 Li Ka Shing Knowledge Institute, St. Michael’s Hospital, Unity Health Toronto, University of Toronto, Toronto, ON M5B 1W8, Canada

**Keywords:** growth differentiation factor 15, prognosis, major adverse limb events, peripheral artery disease

## Abstract

**Background/Objectives:** Peripheral artery disease (PAD) impacts more than 200 million individuals globally and leads to mortality and morbidity secondary to progressive limb dysfunction and amputation. However, clinical management of PAD remains suboptimal, in part because of the lack of standardized biomarkers to predict patient outcomes. Growth differentiation factor 15 (GDF15) is a stress-responsive cytokine that has been studied extensively in cardiovascular disease, but its investigation in PAD remains limited. This study aimed to use explainable statistical and machine learning methods to assess the prognostic value of GDF15 for limb outcomes in patients with PAD. **Methods:** This prognostic investigation was carried out using a prospectively enrolled cohort comprising 454 patients diagnosed with PAD. At baseline, plasma GDF15 levels were measured using a validated multiplex immunoassay. Participants were monitored over a two-year period to assess the occurrence of major adverse limb events (MALE), a composite outcome encompassing major lower extremity amputation, need for open/endovascular revascularization, or acute limb ischemia. An Extreme Gradient Boosting (XGBoost) model was trained to predict 2-year MALE using 10-fold cross-validation, incorporating GDF15 levels along with baseline variables. Model performance was primarily evaluated using the area under the receiver operating characteristic curve (AUROC). Secondary model evaluation metrics were accuracy, sensitivity, specificity, negative predictive value (NPV), and positive predictive value (PPV). Prediction histogram plots were generated to assess the ability of the model to discriminate between patients who develop vs. do not develop 2-year MALE. For model interpretability, SHapley Additive exPlanations (SHAP) analysis was performed to evaluate the relative contribution of each predictor to model outputs. **Results:** The mean age of the cohort was 71 (SD 10) years, with 31% (*n* = 139) being female. Over the two-year follow-up period, 157 patients (34.6%) experienced MALE. The XGBoost model incorporating plasma GDF15 levels and demographic/clinical features achieved excellent performance for predicting 2-year MALE in PAD patients: AUROC 0.84, accuracy 83.5%, sensitivity 83.6%, specificity 83.7%, PPV 87.3%, and NPV 86.2%. The prediction probability histogram for the XGBoost model demonstrated clear separation for patients who developed vs. did not develop 2-year MALE, indicating strong discrimination ability. SHAP analysis showed that GDF15 was the strongest predictive feature for 2-year MALE, followed by age, smoking status, and other cardiovascular comorbidities, highlighting its clinical relevance. **Conclusions:** Using explainable statistical and machine learning methods, we demonstrated that plasma GDF15 levels have important prognostic value for 2-year MALE in patients with PAD. By integrating clinical variables with GDF15 levels, our machine learning model can support early identification of PAD patients at elevated risk for adverse limb events, facilitating timely referral to vascular specialists and aiding in decisions regarding the aggressiveness of medical/surgical treatment. This precision medicine approach based on a biomarker-guided prognostication algorithm offers a promising strategy for improving limb outcomes in individuals with PAD.

## 1. Introduction

Atherosclerotic blockage in the arteries supplying the lower extremities secondary to peripheral artery disease (PAD) impacts over 200 million people worldwide [1,2]. Despite its strong link to catastrophic complications such as limb amputation and increased mortality rates, PAD is often inadequately managed [3]. A major factor contributing to this gap in care is the lack of standardized biomarkers that can accurately identify patients at elevated risk for adverse outcomes and inform treatment decisions [3]. Consequently, there is a critical need for the discovery and validation of biomarkers to improve risk assessment and clinical management of PAD [4].

Growth differentiation factor 15 (GDF15), part of the broader transforming growth factor-beta (TGF-β) superfamily, is a cytokine that becomes upregulated in response to cellular stress [5]. It has been implicated in a range of biological processes, including inflammation, metabolic homeostasis, vascular dysfunction, and atherosclerosis [5]. GDF15 has emerged as a promising biomarker for cardiovascular disease, attracting increasing research interest in recent years [6,7]. Elevated circulating levels of GDF15 have been linked to a heightened risk of coronary artery disease, pathological cardiac remodeling, and the onset of heart failure [8,9]. While its relevance in cardiac disease has been extensively examined, GDF15 remains underexplored in the context of PAD [8,9]. Given the overlapping pathophysiological mechanisms shared by PAD and cardiac disease, including endothelial dysfunction, metabolic dysregulation, and atherosclerosis [10], there is important value in investigating GDF15’s prognostic utility in patients with PAD.

Numerous prior investigations have explored the relationships between specific protein biomarkers and outcomes in PAD, often relying solely on conventional statistical methods and excluding relevant clinical variables from their analyses [11,12,13,14]. However, PAD is a biologically complex condition influenced by a network of metabolic pathways, clinical characteristics, and circulating proteins [15]. Based on this multifactorial nature, we postulated that combining biomarker data with relevant clinical features could substantially improve the accuracy of outcome prediction compared to evaluating protein markers in isolation [15]. In addition, employing explainable machine learning approaches to integrate clinical and biomarker data holds promise for generating accurate, transparent, and clinically relevant models for PAD prognostication [16,17,18].

This study aimed to evaluate the prognostic value of GDF15 for PAD outcomes through explainable statistical and machine learning methods that combine circulating plasma GDF15 levels with clinically relevant characteristics to develop accurate and informative predictive models. Such prognostic models have the potential to enhance early risk assessment in patients with PAD, enabling the timely identification of individuals at elevated risk for adverse events and supporting the implementation of more aggressive medical and/or surgical interventions aimed at preserving limb function and improving clinical outcomes.

## 2. Materials and Methods

### 2.1. Ethics

The Research Ethics Board at Unity Health Toronto approved the study protocol on 8 February 2017 (REB #16-375). Prior to participation, all individuals provided written informed consent. The study was carried out in strict compliance with the ethical standards outlined in the Declaration of Helsinki [19].

### 2.2. Design

The research followed a prognostic study design, and results were presented in line with the TRIPOD+AI reporting standards [20].

### 2.3. Patient Recruitment

Participants were prospectively enrolled between September 2020 and February 2022 from outpatient clinics at our institution. The cohort included patients with PAD only. A diagnosis of PAD was established using a Toe-Brachial Index (TBI) below 0.67 or an Ankle-Brachial Index (ABI) of less than 0.9, along with clinical evidence of reduced or absent pedal pulses [21]. Patients were excluded if they had elevated troponin levels, acute coronary syndrome, or acute limb ischemia within the prior three months.

### 2.4. Baseline Variables

Baseline demographic and clinical information was collected for all participants using their medical records, including age, sex, smoking history (current and former), and the presence of comorbidities such as hypertension, diabetes mellitus, dyslipidemia, coronary artery disease (CAD), congestive heart failure (CHF), and a history of stroke or transient ischemic attack (TIA). Cardiovascular risk factors were classified based on the standards outlined by the American College of Cardiology [22,23]. Hypertension was recognized by either a diastolic pressure of ≥80 mmHg, a systolic blood pressure of ≥130 mmHg, or the use of prescribed antihypertensive therapies [22,23]. Criteria for dyslipidemia included total cholesterol levels above 5.2 mmol/L, triglyceride concentrations exceeding 1.7 mmol/L, or current use of lipid-lowering medications [22,23]. Diabetes was defined as an HbA1c level of 6.5% or greater, or ongoing treatment with glucose-lowering agents [22,23]. Additionally, records were kept of participants’ medications including acetylsalicylic acid (ASA), statins, angiotensin-converting enzyme inhibitors (ACE-I), and angiotensin II receptor blockers (ARB).

### 2.5. Plasma GDF15 Concentration Measurement

At the time of enrollment, blood samples were collected from participants, and plasma concentrations of GDF15 were measured in duplicate using the LUMINEX assay (Bio-Techne, Minneapolis, MN, USA) [24]. Prior to analysis, the MagPix platform (Luminex Corp, Austin, TX, USA) [25] was calibrated using the Fluidics Verification and Calibration bead kits from the same manufacturer [26]. To ensure consistency and minimize variability, all assays were performed on a single day. Both intra- and inter-assay coefficients of variation were kept below 10%. Each sample was analyzed with a minimum of 50 beads, and data processing was carried out using Luminex xPonent software, version 4.3 [27].

### 2.6. Follow-Up and Outcomes

Follow-up outpatient visits were scheduled at one and two years after the initial assessment. The main endpoint of the study was the incidence of major adverse limb events (MALE) within a two-year follow-up period. MALE was defined as a composite outcome that included lower extremity vascular interventions (either open surgical or endovascular revascularization procedures), major amputations above the ankle, or episodes of acute limb ischemia. The latter was characterized by a sudden decrease in limb perfusion lasting fewer than 14 days, resulting from arterial thrombosis or embolic events [28].

### 2.7. Model Development and Evaluation

The predictive modeling approach selected was Extreme Gradient Boosting (XGBoost), a machine learning method that enhances prediction accuracy by integrating numerous decision trees into a single, robust ensemble model [29]. Decision trees segment data into hierarchical branches based on various covariates to generate prediction rules for a given outcome [30]. As a non-parametric method, XGBoost efficiently manages large, complex datasets [29]. It also offers advantages in scalability and speed through parallel computation, alongside built-in regularization to reduce overfitting and enhance model generalizability [29]. The algorithm was selected because of its extensive use in prior studies for both classification and regression analyses, and its proven ability to accurately predict clinical outcomes from structured data [31,32,33].

The data were randomly divided, allocating 70% for model training and the remaining 30% for testing. To predict MALE over two years, the XGBoost algorithm was trained employing 10-fold cross-validation. To reduce overfitting, 10-fold cross-validation was used for model training and an unseen, independent test set was used for model evaluation. To choose the best hyperparameters for the model, an exhaustive grid search was employed. The model incorporated demographic and clinical features, including age/sex, comorbidities such as dyslipidemia, hypertension, diabetes, smoking status, CAD, CHF, history of stroke or TIA, and medication use (ASA, statins, and ACE-I or ARB), as well as plasma GDF15 concentrations. After training, the model’s performance was evaluated on the independent, unseen test set. 

### 2.8. Statistical Analysis

Baseline characteristics were summarized using descriptive statistics. Continuous variables were presented as mean ± standard deviation. Categorical variables were presented as counts (percentages). The primary metric for evaluating model performance was the area under the receiver operating characteristic curve (AUROC). Secondary metrics included accuracy, sensitivity, specificity, positive predictive value (PPV), and negative predictive value (NPV). Youden’s Index was employed to establish the optimal classification cutoff, identifying the threshold that maximizes sensitivity and specificity according to ROC curve analysis [34]. Prediction histogram plots were generated to assess the ability of the predictive model to discriminate between patients who develop vs. do not develop 2-year MALE. SHapley Additive exPlanations (SHAP) analysis was performed to assess the contribution of each feature to the model’s output for explainability [35]. Based on a validated sample size calculator for clinical prediction models, to achieve a minimum AUROC of 0.8 with an outcome rate of approximately 34% and 14 features, a minimum sample size of 453 patients with 155 events is required [36]. Our cohort of 454 patients with 157 primary events met this sample size requirement. Statistical significance was set at a two-sided *p*-value less than 0.05. All statistical analyses, as well as model training and evaluation, were conducted using Python version 3.13.3 [37].

## 3. Results

### 3.1. Patients

The study cohort comprised 454 patients with PAD. All patients had asymptomatic PAD or intermittent claudication. The mean age was 71.11 (SD 9.64) years. The proportion of females was 30.6% (*n* = 139). Cardiovascular comorbidities were prevalent in this cohort, as expected of PAD patients. Hypertension was present in 83.3% of patients and dyslipidemia showed a similar pattern, affecting 82.4% of patients. Diabetes was also common among PAD patients (42.3%). Current smoking was recorded in 34.3% of patients, while past smoking was recorded in 65.7% of patients. Past cardiovascular history was also significant in this cohort. CAD was prevalent in the group (39.2%), and a prior history of TIA or stroke was seen in 18.1% of patients. Additionally, 6.2% of patients had CHF. Medication usage was in line with the risk profile of PAD patients. Statin use was common (76.4%), as was the use of ACE-Is or ARBs (57.5%). Likewise, ASA use was reported in 76.9% of patients (Table 1).

### 3.2. Outcomes

Over the 2-year follow-up period, MALE was reported in 157 patients (34.6%). In total, 92 patients (20.3%) underwent endovascular intervention. Similarly, open intervention was performed in 91 patients (20.0%). Major amputation was reported in 31 patients (6.8%). No patients developed acute limb ischemia (Table 2).

### 3.3. Model Performance for Predicting 2-Year MALE

**Figure 1:**
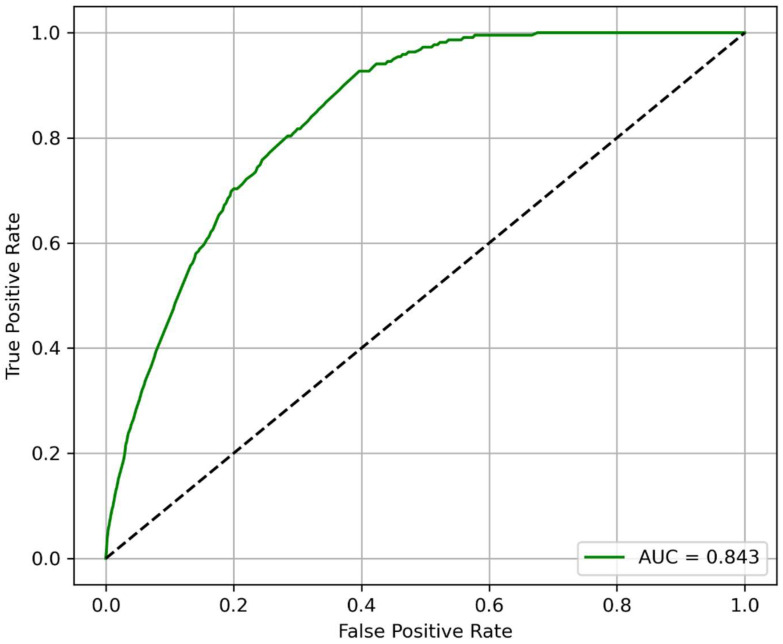
Extreme Gradient Boosting (XGBoost) model receiver operating characteristic curve for predicting 2-year major adverse limb events based on plasma growth differentiation factor 15 (GDF15) levels and demographic/clinical variables. Abbreviation: AUC (area under the receiver operating characteristic curve).

The performance of the XGBoost model for predicting 2-year MALE using circulating GDF15 levels and demographic/clinical features is shown in the ROC curve displayed in Figure 1, achieving an AUROC of 0.843. At the optimal cut-off probability of 0.192 based on Youden’s Index, the model achieved an accuracy of 83.5%, sensitivity of 83.6%, specificity of 83.7%, PPV of 87.3%, and NPV of 86.2%. There was insufficient data to generate a reliable precision-recall curve. The AUROC improved from 0.73 using clinical features alone to 0.84 using both clinical features and GDF15 (*p* < 0.001), demonstrating the prognostic importance of GDF15 for 2-year MALE.

**Figure 2:**
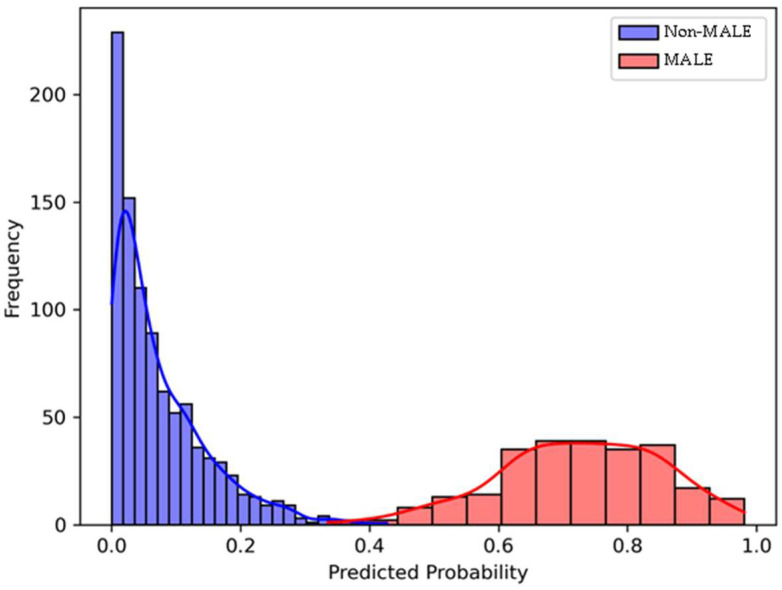
Predicted probability histogram for 2-year major adverse limb events (MALE) using the Extreme Gradient Boosting (XGBoost) model incorporating demographic/clinical features and plasma growth differentiation factor 15 (GDF15) levels. There is a clear separation in predicted risk scores between patients with 2-year MALE (red) and patients without 2-year MALE (blue).

The prediction probability histogram for 2-year MALE showed strong separation between patients who developed MALE and those who did not, based on prediction probabilities generated by the XGBoost model. Among the non-MALE group, predicted probabilities were heavily concentrated near zero, with the highest frequency observed below 0.1. In contrast, patients who experienced MALE had predicted probabilities largely ranging from 0.6 to 0.9, with a peak of 0.75. This clear distinction in predicted probability distributions indicates that the model was highly effective at differentiating between patients who develop vs. do not develop 2-year MALE (Figure 2).

### 3.4. SHAP Analysis of XGBoost Model for Explainability

**Figure 3:**
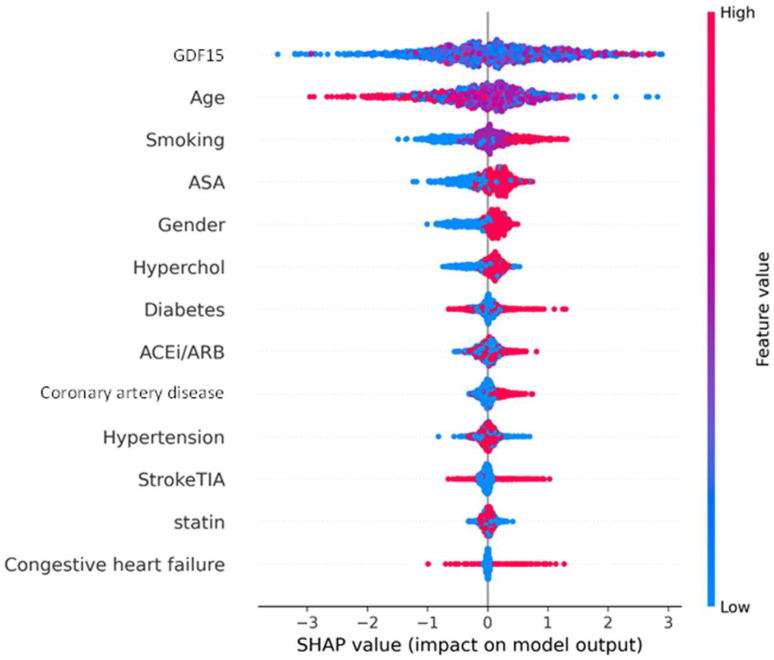
SHapley Additive exPlanations (SHAP) beeswarm plot showing feature contributions to the prediction of 2-year MALE in PAD patients. Each point represents one patient; the position on the x-axis shows the impact on the model’s output, and the color indicates the feature value (red = high, blue = low). Abbreviations: GDF15 (growth differentiation factor 15), ASA (acetylsalicylic acid), hyperchol (dyslipidemia), ARB (angiotensin II receptor blocker), ACEi (angiotensin-converting enzyme inhibitor), StrokeTIA (previous stroke or transient ischemic attack).

The SHAP beeswarm plot for MALE in Figure 3 provides detailed insight into the impact of each predictive feature on the model’s output. GDF15 was the strongest contributor to the prediction of 2-year MALE in PAD patients. High GDF15 values were consistently associated with a positive SHAP value, meaning they increased the model’s probability of predicting the development of 2-year MALE. The SHAP values for GDF15 extended as high as +3, indicating a very strong effect on 2-year MALE prediction. Age was the second most influential feature, with older patients also shifting predictions toward higher MALE risk, as expected. Smoking showed a spread of influence, with current/past smoking increasing risk in many individuals. Other features, such as ASA use, sex, and diabetes, showed moderate contributions, while features like previous stroke/TIA, CHF, and statin use had relatively small impacts, with SHAP values clustering around zero. In our study population, there were no independent determinants of GDF15.

**Figure 4:**
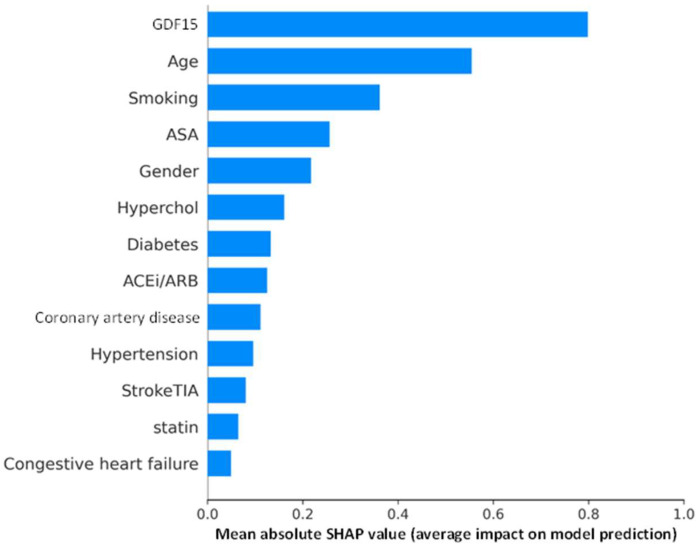
SHapley Additive exPlanations (SHAP) bar plot displaying mean absolute SHAP values for each feature in the 2-year major adverse limb event prediction model. Abbreviations: GDF15 (growth differentiation factor 15), ASA (acetylsalicylic acid), hyperchol (dyslipidemia), ARB (angiotensin II receptor blocker), ACEi (angiotensin-converting enzyme inhibitor), StrokeTIA (previous stroke or transient ischemic attack).

The SHAP summary plot in Figure 4 quantifies the average importance of each feature in the prediction model by calculating the mean absolute SHAP values. GDF15 had the highest average SHAP value at 0.798, indicating that it was the most influential predictor for 2-year MALE. This was followed by age (0.554) and smoking (0.361), both contributing substantially to the model’s output. ASA use (0.257) and sex (0.218) were also important. Other clinical variables such as dyslipidemia (0.161), diabetes (0.132), ACE-I/ARB use (0.125), and CAD (0.111) showed moderate contributions. The remaining features, including hypertension (0.096), previous stroke/TIA (0.080), statin use (0.064), and CHF (0.050), had smaller impacts on the model’s predictions.

## 4. Discussion

### 4.1. Summary of Findings

In this study, we applied explainable statistical and machine learning techniques to identify GDF15 as a circulating biomarker strongly associated with limb outcomes in patients with PAD. By integrating GDF15 levels with demographic and clinical data, we constructed a highly accurate prognostic model for predicting 2-year MALE in individuals with PAD (AUROC 0.84). We showed that this model distinctly separated patients who developed 2-year MALE from those who did not through a prediction probability histogram, highlighting the potential clinical utility of this biomarker-based model. Furthermore, we demonstrated the importance of GDF15 in prognosticating PAD outcomes through SHAP analysis, which showed that this biomarker was the most important predictive feature in the model, above other clinically important variables such as age, smoking status, and cardiovascular comorbidities. Collectively, these results emphasize the value of GDF15 for risk stratification in PAD, potentially guiding targeted medical and surgical interventions to improve limb outcomes among high-risk individuals. Given these promising prognostic capabilities, further basic and translational studies are needed to elucidate GDF15’s biological mechanisms in PAD pathogenesis and to assess its potential as a therapeutic target for reducing adverse limb events.

### 4.2. Comparison with Previous Studies

Prior studies have examined GDF15’s role in PAD. De Haan and colleagues (2017) showed that GDF15 was linked to mortality and limb loss in PAD patients [11]. There were several key differences in our studies. First, while De Haan and colleagues primarily recruited patients with chronic limb threatening ischemia, our cohort consisted primarily of patients with intermittent claudication, a less severe form of PAD [11]. Patients with chronic limb threatening ischemia generally have severe PAD requiring urgent intervention, and the opportunity to prevent limb loss through aggressive risk reduction strategies is limited [11]. By focusing on patients with less severe PAD, our study aims to develop earlier risk-stratification strategies to prevent limb loss. Second, while De Haan and colleagues focused on assessing GDF15 as an individual biomarker using traditional statistical techniques, we used explainable machine learning to combine GDF15 with clinically relevant factors to develop a highly accurate predictive model for 2-year MALE [11]. Therefore, while De Haan and colleagues showed an association between GDF15 and major amputation/death, we developed an accurate and informative biomarker-based prognostic machine learning model with greater clinical applicability potential [11]. Finally, while De Haan and colleagues only assessed major amputation and death, we evaluated the more clinically relevant outcome of MALE, which additionally incorporates the need for open or endovascular revascularization and the development of acute limb ischemia [11]. Given that these are clinically important limb events in patients with PAD, our findings have greater potential for clinical relevance. Altogether, our study corroborates the existing literature and adds to the knowledge base through the development of explainable machine learning models that combine circulating GDF15 levels with clinically relevant factors to accurately prognosticate limb outcomes in patients with PAD. These findings highlight the necessity for additional mechanistic research to better understand GDF15’s role in the development and progression of PAD. Such insights could ultimately enhance risk stratification and lead to innovative, targeted treatments aimed at improving limb outcomes in patients with PAD.

### 4.3. Explanation of Findings

Several potential mechanistic pathways may explain the link between elevated GDF15 levels and increased MALE risk in patients with PAD. At the cellular level, GDF15 expression is increased within subendothelial macrophages in human atherosclerotic plaques, where it colocalizes with oxidized low-density lipoprotein containing macrophages and various inflammatory mediators [38]. In the context of myocardial injury, GDF15 expression is markedly upregulated in infarcted myocardium compared to healthy tissue and is elevated in the bloodstream following acute myocardial infarction [39]. Experimental models in mice demonstrate a nearly 20-fold increase in myocardial GDF15 mRNA following myocardial infarction relative to uninjured hearts [40]. Clinically, elevated GDF15 levels have been observed in individuals with vascular dysfunction, atherosclerosis, and thrombotic conditions [41]. Additionally, GDF15 serves as a predictor of chronic kidney disease progression and renal function decline in patients with existing kidney pathology [42]. The broad involvement of GDF15 in diverse cardiovascular and systemic conditions, involving inflammation, metabolic dysregulation, atherosclerosis, and endothelial impairment, likely underpins its strong prognostic association with adverse limb events in PAD patients, as observed in our study [43].

### 4.4. Implications

Our findings provide important clinical insights for managing patients with PAD. Measuring plasma GDF15 concentrations allows clinicians to more precisely stratify PAD patients by their risk of MALE. This biomarker-driven strategy is particularly valuable in primary care settings, where early identification of high-risk individuals enables targeted management pathways [44]. Primary care physicians could incorporate GDF15 measurement into standard blood tests for patients with PAD, enabling enhanced risk stratification [44]. By combining GDF15 levels with clinical features in a predictive machine learning model, our approach offers practical utility for guiding clinical decisions related to MALE risk. Patients identified as high risk should be promptly referred to vascular surgery for further evaluation [45]. In contrast, patients identified as low risk can remain under the care of their primary physician, concentrating on the optimization of cardiovascular risk factors via antiplatelet agents, statins, and lifestyle changes [46]. For individuals referred to specialist care, our model facilitates treatment plans tailored to their risk profile. Vascular surgeons can use this tool alongside clinical judgment to identify patients at elevated risk for adverse limb outcomes who could benefit from: (1) further vascular imaging to evaluate anatomical details and disease severity [47], (2) low-dose rivaroxaban therapy [48], and/or (3) surgical interventions aimed at limb preservation [49,50]. Importantly, although our model showed strong separation between patients who develop vs. do not develop MALE, there is still considerable overlap between individuals. Therefore, careful clinical assessment and judgment in conjunction with evaluating model outputs is necessary to provide appropriate clinical care. Overall, this predictive model holds promise for enhancing the management of PAD in both primary and specialized care by enabling more effective screening, precise risk stratification, and timely identification of individuals at elevated risk for limb-related complications. This strategy could help minimize unnecessary referrals to specialists while improving patient outcomes [51]. Integrating our GDF15-based model into clinical practice can enhance personalized risk assessment, optimize referral decisions, and enable tailored treatments, ultimately supporting better cardiovascular outcomes and more efficient use of healthcare resources [52]. Additionally, given that we have demonstrated the prognostic importance of GDF15 in PAD, the use of GDF15 as a therapeutic target in patients with PAD is an important avenue for further investigation to potentially improve limb outcomes [53].

### 4.5. Limitations

Several limitations of this study should be acknowledged when interpreting the findings. First, as this study was performed at a single academic institution, the extent to which these results apply to broader, more diverse populations remains uncertain. Therefore, multicenter validation is essential to verify the generalizability of our findings. Second, the follow-up duration was limited to two years. Considering the chronic, progressive nature of PAD, longer-term studies are necessary to evaluate the long-term prognostic utility of GDF15. Given that a significant proportion of our PAD cohort had existing CAD, future studies with more restrictive cohorts of PAD patients only (without CAD) may provide better interpretability of the prognostic value of GDF15 specifically in PAD populations. Additionally, although most clinically relevant variables were captured, several factors, including chronic kidney disease, obesity, and beta blocker usage, were not recorded. Future studies that include these variables may strengthen model performance and clinical applicability. Furthermore, GDF15 plasma concentrations were measured at one timepoint during enrollment, which does not allow for the investigation of the impact of changes in GDF15 concentrations on the occurrence of MALE. Additionally, the time elapsed since blood sampling and the occurrence of MALE was not available for analysis. Future studies with the necessary data to characterize event occurrence in relation to time elapsed since blood sampling, and models that consider time as a covariate, would allow for a more comprehensive analysis of the prognostic value of GDF15 in PAD. While we have extensively verified our results by assessing multiple validated performance metrics, creating a predicted probability histogram, and performing SHAP analysis, future validation studies using classical multivariable analysis with traditional statistical techniques would be prudent. Third, the sample size was insufficient to robustly assess relationships between biomarkers and individual components of MALE. Future investigations involving larger patient cohorts and increased event rates are needed to determine the predictive performance of these biomarkers for specific limb-related outcomes. Additionally, the small sample size may reduce model efficacy, which could be improved with larger cohorts in future studies. Lastly, GDF15 testing can be performed using commercially available assays such as LUMINEX (Bio-Techne, Minneapolis, MN, USA) and is therefore feasible for most laboratories [24]. However, GDF15 testing is currently predominantly restricted to research environments; thus, further translational and implementation research is required to evaluate its practicality, cost-effectiveness, and integration into standard clinical workflows for patients with PAD.

## 5. Conclusions

This study employed explainable statistical and machine learning techniques to identify GDF15 as a prognostic biomarker for 2-year MALE in patients with PAD. By integrating GDF15 levels with clinically relevant features, we developed an accurate and informative prognostic model for PAD outcomes. This GDF15-based model has the potential to enhance risk stratification and enable personalized management strategies within the PAD population. Early identification of high-risk patients facilitates timely, intensified interventions, including referrals to multidisciplinary specialists and aggressive medical/surgical therapies to prevent limb loss. Furthermore, our findings highlight the need for further basic and translational research to elucidate the mechanistic pathways by which GDF15 influences PAD pathophysiology, which may facilitate the development of novel targeted therapies to improve limb outcomes.

## Figures and Tables

**Table 1:** Baseline characteristics.

	Patients with PAD (*n* = 454)
Age, years	71.11 ± 9.64
Female sex	139 (30.6)
Hypertension	378 (83.3)
Dyslipidemia	374 (82.4)
Diabetes	192 (42.3)
Smoking, past	241 (65.7)
Smoking, current	126 (34.3)
Congestive heart failure	28 (6.2)
Coronary artery disease	178 (39.2)
Previous stroke or transient ischemic attack	82 (18.1)
Statin	347 (76.4)
ACE-I/ARB	261 (57.5)
ASA	349 (76.9)

Values are presented as mean ± standard deviation for continuous variables and frequency (percentage) for categorical variables. Abbreviations: PAD—peripheral artery disease; ARB—angiotensin II receptor blocker; ACE-I—angiotensin-converting enzyme inhibitor; ASA—acetylsalicylic acid.

**Table 2:** Outcomes over 2 years of follow-up.

	Patients with PAD (*n* = 454)
Major adverse limb event	157 (34.6)
Vascular intervention	
Endovascular	92 (20.3)
Open	91 (20.0)
Major amputation	31 (6.8)
Acute limb ischemia	0 (0)

Values are presented as frequency (percentage). Abbreviation: PAD (peripheral artery disease).

## Data Availability

The original contributions presented in the study are included in the article; further inquiries can be directed to the corresponding author.
